# Ovarian tissue vitrification and heterotopic autologous transplantation in prepubertal Wistar rats

**DOI:** 10.5935/1518-0557.20180019

**Published:** 2018

**Authors:** Leticia Wietcovsky, David Til, Rafael Alonso Salvador, Nicole Louise Lângaro Amaral, Alfred Paul Senn, Vera Lucia Lângaro Amaral

**Affiliations:** 1 Laboratório de Biotecnologia da Reprodução (LBR), Universidade do Vale do Itajaí (UNIVALI), Itajaí, Santa Catarina, BrazilLaboratório de Biotecnologia da Reprodução (LBR), Universidade do Vale do Itajaí (UNIVALI), Itajaí, Santa Catarina, Brazil; 2 Centro Clínico Veterinário (CCV), Itajaí, Santa Catarina, Brazil

**Keywords:** cryopreservation, ovarian tissue, vitrification, autologous heterotopic transplantation, restoration of endocrine function

## Abstract

**Objective:**

To evaluate the efficiency of ovarian tissue heterotopic autografting after vitrification in prepubertal rats.

**Methods:**

Fragments of excised ovaries from prepubertal rats were used after assessing post-warming cellular viability, to determine the best vitrification protocol prior to retroauricular autografting. Pre-pubertal females (N=24) were castrated and divided into three group: Group 1 - fresh ovarian tissue transplantation; Group 2 - vitrified/warmed tissue transplantation; Group 3 - bilateral oophorectomy without transplantation. The ovarian fragments were exposed to solutions from the Ingamed^®^ commercial kit, allocated in bacteriological loops and immersed in liquid nitrogen. Sixty days after transplantation, a vaginal mucus sample was collected for cytology tests, followed by sacrificing the animal, performing a cardiac puncture for collecting a blood sample to determine luteinizing hormone and estradiol levels, and excision of the transplanted fragment for histology tests.

**Results:**

Vaginal cytology revealed that 87.5% of females from groups 1 and 2 had estrus while all females in Group 3 remained in diestrus. The mean LH value in groups 1 (0.08 mIU/mL) and 2 (0.34 mIU/mL) were statistically different from that of Group 3 (2.27 mIU/mL). E2 values did not differ between the groups. The histological analysis of Group 1 excised grafts versus those from Group 2 showed a higher percentage of primary follicles (62.5% vs. 12.5%), developing follicles (75% vs. 25%), corpus luteum (37.5% vs. 12.5%) and stromal region (100% vs. 87.5%).

**Conclusion:**

This study indicated that pre-pubertal ovarian tissue vitrification can be used to preserve fertility and to restore endocrine function in castrated rats.

## INTRODUCTION

Infertility, which affects the human population worldwide, has significant psychological consequences (Brezina *et al*., 2015), and it is considered a public health problem by the World Health Organization (WHO) (Van der Poel, 2012). Among the causes of infertility, neoplasia and their associated treatments, such as chemotherapy and radiotherapy, exert irreversible cytotoxic effects on male and female gonads, which eventually lead to hormonal and reproductive failure (Schunemann Jr *et al*., 2011; Almodin & Costa, 2014). Considering that the estimated 2016-2017 occurrence of cancer in Brazil will be around 600,000 new cases (INCA, 2015), equally divided between men and women, and that anticancer treatments are increasingly efficient, it becomes necessary to offer these patients the possibility to preserve their fertility. According to the recommendations from the American Society of Clinical Oncology, some of the proven effective methods currently available for preserving female fertility are embryo cryopreservation, oophoropexy prior to localized radiotherapy, and oocyte cryopreservation (Loren *et al*., 2013).

For many oncologists, cryopreservation of oocytes and embryos may be unfeasible, mainly due to the need for hormonal stimulation to obtain oocytes and the ensuing delayed cancer treatment onset. Furthermore, it is not usable in young patients who have not initiated puberty or to adult women who suffer from hormone-dependent cancer (Moura *et al*., 2015). Alternatively, the association of cryopreservation and ovarian tissue reimplantation can be performed at any time during the menstrual cycle and does not require hormonal stimulation (Donnez *et al*., 2004). This technique is still new for young cancer patients, as the first live birth after cryopreserved ovarian tissue before menarche followed by transplantation at adulthood occurred only recently (Demeestere *et al*., 2015).

There are two main cryopreservation techniques: the so-called slow freezing method, which requires gradual cooling ramps and low concentrations of cryoprotectants, and the vitrification method, that consists in ultrafast cooling of the biological material in the presence of high concentrations of cryoprotective agents (Niemann, 1991; Castro *et al*., 2011). The slow freezing technique has been routinely used for ovarian tissue cryopreservation (Newton *et al*., 1996; Oktay & Karlikaya, 2000), however, the vitrification technique has been shown to have equal or greater efficiency in ovarian tissue preservation than the slow freezing protocol (Silva, 2014). The first success with a live birth in humans was obtained with the slow freezing technique and orthotopic transplantation of cryopreserved ovarian tissue in an adult patient (Donnez *et al*., 2004).

The transplantation can be classified according to the implantation site, as orthotopic, in which the tissue is transplanted to its original site, or heterotopic when the tissue is transplanted to a region other than its original one (Sonmezer & Oktay, 2004). The first one enables a possible spontaneous gestation; whereas heterotopic transplantation requires the use of ancillary techniques, such as *in-vitro* culture of ovarian follicles followed by oocyte maturation and *in-vitro* fertilization, enabling embryo production and transfer (Lunardi *et al*., 2013). Several studies have been carried out to try to find the best place to perform ovarian auto-transplantation (d'Acampora *et al*., 2004; Donnez *et al*., 2013). Experimentally, we may consider sites such as: underneath the renal capsule (Oktay & Karlikaya, 2000), the retroperitoneum (d'Acampora *et al*., 2004) in rats, subcutaneously (Scalercio *et al*., 2015) and iliac fossa and omentum (Igarashi *et al*., 2010) in non-human primates. 

To date, about 86 live births have been recorded in the literature, either in peer-reviewed journals or in conference proceedings (Jadoul *et al*., 2017). Among these, only two births came from vitrified ovarian tissue (Suzuki *et al*., 2015), probably because vitrification is a more recently established technique, and there was no time to perform a transplantation in a clinically healthy patient after oncological treatment within the considered period.

Several techniques can be used to evaluate the competence of the ovarian graft post-cryopreservation. In addition to DNA fragmentation, the technique usually employed is the morphological analysis of the tissue through classical histology by staining with hematoxylin-eosin, due to its low cost and ease of execution. In a smaller scale, fluorescence and electron microscopy, cytological analysis of vaginal smears in animals, serum levels of pituitary and gonadal hormones, and follicular in vitro culture are also used (Rodriguez-Wallberg & Oktay, 2014).

With the increase in the number of specialized centers providing care to women in search of alternatives for fertility preservation, the development of more effective protocols is fundamental to successfully put into practice ovarian tissue transplantation and cryopreservation techniques. Therefore, the objective of this study is to evaluate heterotopic autografting of pre-vitrified ovarian tissue efficiency using prepubertal rats as experimental models.

## MATERIALS AND METHODS

### Animals

For this study, 31 female rats (Wistar) 30-60 days old were obtained from the vivarium of the University of "Vale do Itajaí - UNIVALI, SC". This study was approved by the Ethics Committee on Animal Use (CEUA) under number 003/16.

### Vitrification protocols

To determine the best vitrification protocol, 18 ovarian fragments from Wistar rats (Rattus norvegicus) were obtained from 7 females in pre-pubertal age (30 to 60 days). After sacrifying the animals in a CO_2_/O_2_ chamber, oophorectomy was performed and the ovaries were cut into 1mm-thick fragments. One of the dissected fragments was submitted to follicular viability using Trypan blue (Sigma, São Paulo, Brazil) vital stain (0.4%) (Fauque *et al*., 2007). Unstained follicles were considered viable. The other fragments were divided and submitted to three vitrification protocols.

Protocol 1: the fragments were exposed for 25 minutes to the vitrification solution (VS1), composed of 7.5% dimethylsulfoxide (DMSO, Nuclear, São Paulo, Brazil) and 7.5% ethylene glycol (EG, Sigma, São Paulo, Brazil), and subsequently to a vitrification solution (VS2) containing 20% DMSO, 20% EG and 0.4M sucrose (Sigma, São Paulo, Brazil) for 15 minutes. Fragment warming was carried out using 1M and 0.5M sucrose for 1 and 5 minutes respectively, followed by 10 minutes in Modified-HTF (Irvine Scientific^®^, Santa Ana, USA) (adapted from Kagawa *et al*., 2009).

Protocol 2: the fragments were exposed to the same VS1 solution used in protocol 1, but for 10 minutes only, followed by VS2 vitrification solution containing 15% DMSO, 15% EG and 0.4M sucrose for 2 minutes. For warming purposes, the fragments were exposed to 1M, 0.5M and 0.25M sucrose for 5 minutes each (adapted from Chen *et al*., 2006).

Protocol 3: the fragments were exposed to a balancing VI-1 solution containing 7.5% EG and 7.5% DMSO in buffered medium, supplemented with 20% synthetic serum for 25 minutes (Ingamed^®^ Trade Kit, Maringá, Brazil). The fragments were then transferred to a vitrification solution (VI-2), composed of 15% EG, 15% DMSO plus 0.5M sucrose, in buffered medium, supplemented with 20% synthetic serum for 15 minutes. Warming was performed with a DV-1 solution, containing 1M sucrose and 20% synthetic serum in buffered medium for 5 minutes, followed by DV-2, containing 0.5M sucrose and 20% synthetic serum in buffered medium for 5 minutes. Finally, the fragments were exposed to a balancing DV-3 solution, composed of buffered medium and 20% synthetic serum for 10 minutes (adapted from Wang *et al*., 2008).

**Figure 1:**
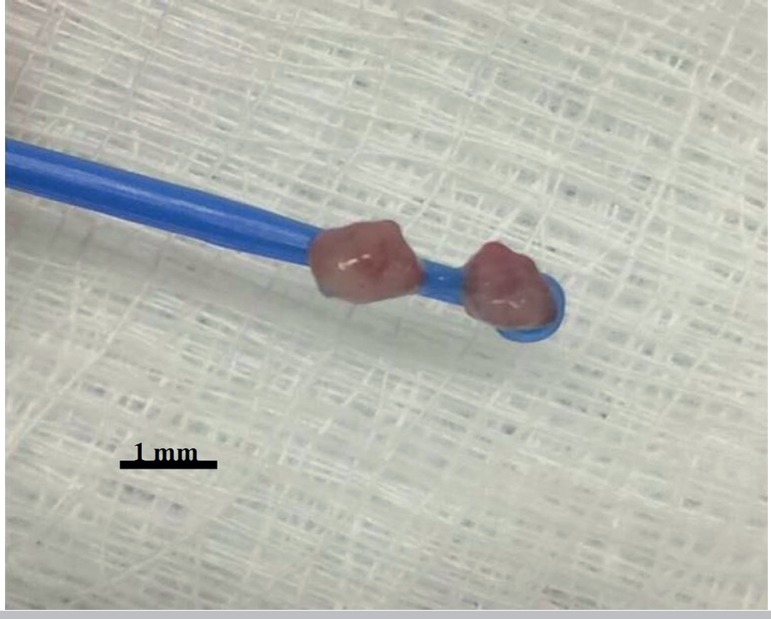
Bacteriological loop used to support the ovarian fragments at the time of vitrification

Vitrification: The whole vitrification procedure was performed on an ice sheet, so that the temperature remained between 6°C and 8°C (Amorim *et al*., 2009). Vitrification was performed using sterile 230 mm bacterial inoculation loops, which were reduced in length to about 2cm (Olen^®^, Teratec, São Paulo, Brazil). The balanced fragments were deposited inside the loop and immediately immersed in liquid nitrogen (-196°C) (Figure 1).

Warming: The first devitrification solution from each protocol was kept at 37°C and the others at room temperature (22±2°C), according to the above-mentioned protocols. After warming, the cell viability was again checked using the vital stain trypan blue.

### Autologous and heterotopic transplantation

Protocol 3 was used to vitrify the ovarian fragments, prior to transplantation, because this protocol yielded the best recovery of viable cells (see Results).

The pre-pubertal Wistar female rats were divided into 3 groups of 8 animals. Group 1: transplantation of fresh ovarian graft in the retroauricular region. Group 2: transplantation of vitrified/warmed ovarian tissue to the retroauricular region. Group 3: bilateral oophorectomy without ovarian tissue reimplantation. For oophorectomy, the animals were anesthetized with a combination of 10% ketamine hydrochloride (40mg/kg) + 2% xylazine hydrochloride (20mg/kg) + 1% acepromazine (3mg/kg) intraperitoneally (IP). For the animals in Group 1, the fresh fragment of the removed ovarian tissue was transferred immediately to the retroauricular region, through an incision of approximately 50mm, with insertion of the graft and subsequent access cauterization. For animals in Group 2, reimplantation was performed in the same way as in Group 1, but after 20 days of oophorectomy and vitrification/warming of the fragments. After the surgical procedures in the groups, 1% ketoprofen analgesic (5mg/kg) was administered subcutaneously. After surgery, the animals were kept in their usual conditions for 60 days. The animal health was ascertained during the handling of the habitats, feeding, and cleaning of the cages.

**Figure 2:**
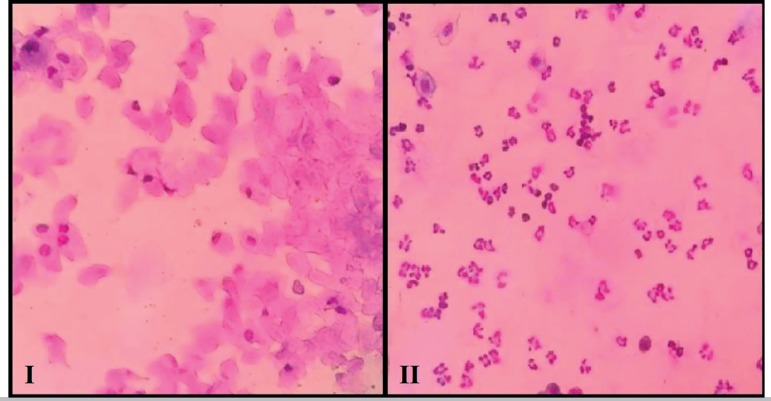
Examples of various stages of vaginal histology observed after transplantation. **I - Estrous stage:** keratinized cells typical of this stage (400x); **II - Diestrous stage:** leukocytes and nucleated cells typical of this stage (400x).

During the four days prior to slaughtering, vaginal cytology was performed to evaluate the estrous cycle phase, washing the vaginal canal with saline daily. The smear was stained with the panoptic kit (RenyLab^®^, Barbacena, MG, Brazil). The presence or absence of leukocytes, nucleated and keratinized cells (Figure 2) was observed to characterize each phase of the estrous cycle; i.e., diestrus, proestrus, estrous and metestrus (Marcondes *et al*., 2002). The animals were killed in a CO_2_/O_2_ chamber and a cardiac puncture was performed for blood collection and determination of serum Luteinizing hormone (LH) and estradiol (E2) levels.

The ovarian fragments were removed from the graft sites and submitted to histological analysis with hematoxylin and eosin staining. The slides with the histological sections were analyzed morphologically and for the presence or absence of primary follicles, developing follicles, corpus luteum, pyknosis and stromal region.

### Hormone analysis

LH and E2 were analyzed by the Laboratório Escola de Análises Clínicas (LEAC/UNIVALI) using chemiluminescence methods (ADVIA-Centaur, Brazil).

### Statistical analysis

The data were submitted to statistical analysis (Instat, GraphPad Software, USA) using ANOVA and Tukey's multiple mean comparison among the three groups. The significance level was set at p<0.05.

## RESULTS

In a preliminary phase, three vitrification protocols were tested in order to optimize the concentration of the cryoprotectants (DMSO, EG, sucrose) and the exposure time to these compounds during the pre- and post-vitrification processes. Cell viability using trypan blue staining was used to determine which of these protocols yielded the highest survival rates. Cell viability after vitrification with protocols 1, 2 and 3 were 81.3%, 90.8% and 96.3%, respectively (data not shown). On this basis, protocol 3 was used for the transplantation experiments.

Sixty days after the surgical transplantation of the ovarian tissue, the estrus cycle phase was determined in the transplanted (Group 1 and Group 2) and non-transplanted castrated females (Group 3) on the day of slaughtering. Vaginal swabs were collected from all females immediately after death. The stained vaginal smears were examined under 400x magnification and the stage of the estrous cycle determined. In both Groups 1 and 2, the presence of an estrous cycle was confirmed in 7 out of 8 females (87.5%) (Figure 2, I-II). All females from Group 3, which only had their ovaries removed and were not transplanted, remained in the diestrus stage (Table 1).

**Table 1:** Estrus cycle phase on the last day of collection of vaginal cytology and individual serum LH (mIU/mL) and E2 (pg/mL) values in the three groups. M: metestrus, D: diestrus, E: estrous, P: proestrus.

	Group 1				Group 2				Group 3		
	Phase	LH	E2		Phase	LH	E2		Phase	LH	E2
**1**	M	0.07	19.49		M	0.56	26.95		D	2.1	11.8
**2**	D	0.07	11.8		M	0.6	20.33		D	1.33	23.95
**3**	E	0.16	13.17		E	0.07	17.69		D	*	*
**4**	E	0.07	*		D	0.78	30.45		D	2.63	11.8
**5**	P	0.07	42.34		M	0.08	20.75		D	2.68	11.8
**6**	M	0.07	24.53		M	0.07	25.41		D	5.43	11.8
**7**	P	0.07	31.91		E	0.41	21.9		D	0.75	15.76
**8**	M	*	*		M	0.13	55.88		D	0.96	13.05

* Analysis not performed due to insufficient serum for analysis.

LH and E2 levels were also measured in each animal and the results are presented in Tables 1 and 2. In groups 1 and 2, the LH levels were ≤0.16 mIU/mL and ≤0.78 mIU/mL, respectively. In Group 3, the LH levels were higher: between 0.75 mIU/mL and 5.43 mIU/mL. Conversely, E2 levels were higher in Groups 1 and 2, with mean values of 23.9±4.8 pg/mL and 27.4±4.3 pg/mL, respectively; about twice as much as what was measured in Group 3: 14.3±1.7 pg/mL (Table 2).

**Table 2:** Mean ± SEM of LH and E2 measured in the serum at the time of slaughtering in the three groups.

Groups	LH (mUI/mL)	E2 (pg/mL)
**1**	0.08±0.01^a,c^	23.87±4.78
**2**	0.34±0.10^a,b^	27.42±4.32^d^
**3**	2.27±0.60^b,c^	14.28±1.70^d^

^a^ *p* < 0.05, ^b,c^ *p* < 0.02, ^d^ *p* < 0.02

**Figure 3:**
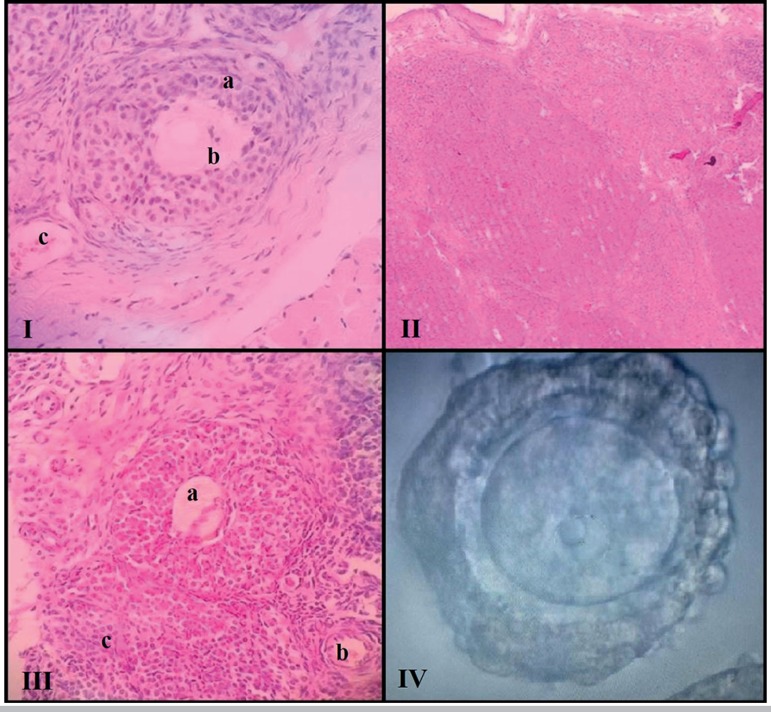
Example of various stages of follicular development. **I -** Pycnosis (a), developing follicle (b), vascularization (c); **II -** Corpus luteum; **III -** Developing follicle (a), primary follicle (b), stroma (c); **IV -** Prophase I oocyte.

In the histological analysis of the grafted tissue, the presence or absence of pycnosis (Figure 3-I), corpus luteum (Figure 3-II), primary follicle (Figure 3-III), developing follicle (Figure 3- III) and stroma (Figure 3-III) were considered. The analysis of the histological sections of Group 1 vs Group 2 showed a higher percentage of primary follicles (62.5% vs. 12.5%), developing follicles (75% vs. 25%), corpus luteum (37.5% vs. 12.5%) and stromal region (100% vs. 87.5%). These two groups also showed pycnotic nuclei, not found in the histological sections of the fragments analyzed before the tissue was re-implanted (Table 3).

**Table 3:** Histological characteristics (in %) of the ovarian fragments (8 per group) of groups 1(fresh/grafted), 2 (vitrified/grafted) e 2*(vitrified/warmed).

Group	Pyknosis	Primary follicle	Developing follicle	Corpus luteum	Stroma
**1**	100% (8/8)	100% (8/8)	75% (6/8)	37.5% (3/8)	100% (8/8)
2*	0 (0/8)	87.5% (7/8)	100% (8/8)	0 (0/8)	100% (8/8)
**2**	87.5% (7/8)	12.5% (1/8)	25% (2/8)	12.5% (1/8)	87.5% (7/8)

In addition, in all histological sections that confirmed the presence of some ovarian structure, it was possible to identify graft neovascularization (Figure 3-III). A vitrified graft fragment was surgically removed after 60 days of transplantation and, after dissection, it revealed the presence of developing follicles containing oocytes with good morphological quality (Figure 3-IV).

## DISCUSSION

Due to the high complexity of the ovarian structure, tissue survival depends not only on the cooling rates during the cryopreservation process but also on thawing and the removal of cryoprotectants (Thomaz *et al*., 2005). In this context, several protocols were tested with the objective of improving the efficiency of ovarian tissue cryopreservation. Three vitrification protocols were tested, the one that demonstrated the greatest cell viability after devitrification was used for the subsequent heterotopic autograft. Viability was tested using trypan blue, a dye used to evaluate cell membrane integrity; i.e., stained follicles have damaged membranes (Jewgenow, 1998). The same dye was used to evaluate the toxicity of cryoprotectants in sheep, demonstrating greater follicular viability when EG and DMSO cryoprotectants were used (Santos *et al*., 2006). In human ovary fragments, the viability of primordial and primary follicles was lower after freezing (71.9%) than before the procedure (87.3%) (Fauque *et al*., 2007). In addition, in fresh tissue, a follicular death of 12.7% was associated with the mechanical trauma caused by tissue sectioning. In our study, this mechanical damage occurred to a lesser extent and was estimated at 3.67%. An increasing exposure time to propanediol (PROH) favors a decrease in the number of viable follicles using slow freezing, an indication that there is a limit to cell dehydration and to metabolic resistance against the toxic effects of the cryoprotectants (Gonçalves *et al*., 2012). Confronting our results to those published in the literature, it may be concluded that the decline in follicle viability found in our study can be considered normal.

In our study, the 25-minute exposure to the solutions containing DMSO and EG at 7.5% provided good tissue permeation and a better recovery of living cells after warming. On the other hand, shorter times in the equilibrium solution did not enable adequate permeation.

For the vitrification technique to work well, it is important that the volume of the vitrification solution that surrounds the fragment be as small as possible when entering liquid nitrogen (Wang *et al*., 2008). In this context, the fragment packaging method interferes with the cryopreservation technique efficacy. Thomaz *et al*. (2005), using rabbit ovaries, introduced the sections of tissue in straws using the slow freezing protocol, and found the survival of only primordial follicles. Using the same animal model, Almodin *et al*. (2004) vitrified the ovarian fragments in cryogenic tubes and obtained spontaneous pregnancies in all animals in which the cryopreserved tissue reimplantation was performed. Wang *et al*. (2008) were successful using human ovary cortex and rat ovary when they allocated the fragments into acupuncture needles, to maximize and simplify the vitrification process. In this study, the ovarian fragments were placed in bacteriological loops and stored in cryogenic tubes, obtaining a rate of 100% recovery of the fragments after thawing. In addition, the easy handling and reduction of the excess volume of the vitrification solution, essential for the success of the technique, have been verified. After reviewing the literature on the subject, no study was found mentioning the type of device used in our study. We believe this type of packaging is a new viable option for cryopreservation of ovarian tissue.

Considering the graft experiments, several studies were carried out in an attempt to find the best anatomical site to perform the ovarian implant (Oktay & Karlikaya, 2000; d'Acampora *et al*., 2004; Igarashi *et al*., 2010). According to Donnez *et al*. (2013), negative aspects in the use of heterotopic transplantation are the differences in temperature, pressure, and vascularization between the heterotopic site and the donor area. In this study, heterotopic transplantation was performed in the retroauricular region, which facilitates visualization of the graft and has adequate vascularization. In a long-term study involving women, endocrine recovery after heterotopic transplantation was demonstrated by the return of hormonal function using vaginal cytology in 87.5% of the women (Kim, 2012).

In rodents, the study of vaginal cytology is effective in evaluating ovarian function due to short estrus periods (4-5 days) and easy cell characterization (Jafarey & Jaffri, 2016). In each phase of the estrous cycle, there are characteristic cells and this change is due to the influence of the hormones that are active in each phase. In the diestrus phase, identified in all the females of Group 3, a higher level of LH and a lower level of E2 were found when compared to the other groups. The different LH values recorded for the different females can be justified by the pulsatile form of release of this hormone (Kim, 2012). However, E2, a marker of ovarian function, is 90% synthesized by the follicles, but it can also be produced through an extra-glandular conversion of testosterone and androstenedione (Saraiva *et al*., 2010). The insufficient production of E2 at this stage explains the lack of negative feedback and the increased LH production. On the other hand, E2 levels were higher in ovarian transplant groups, demonstrating that there was ovarian activity, since E2 is synthesized within the developing follicles and these were present in the histological analysis. Hormone levels are usually restored when the vascular network of the implant is already established and a percentage of tissue remains viable with active secretory cells.

Ovarian transplantation aims to recover hormonal and reproductive functions, and may be an alternative for menopausal women who have cryopreserved their ovarian tissue during their reproductive age. However, transplantation of the tissue fragments is the main factor contributing to the loss of a considerable number of follicles and it is directly related to the time necessary for the reestablishment of blood supply, a delay which may compromise graft survival. The longer the time to angiogenesis, the shorter the graft survival (Jafarey & Jaffri, 2016). The greatest difficulty is in the immediate post-reimplantation period, when the risk of ischemia is higher leading to irreversible follicular loss (Newton *et al*., 1996). Messias (2016), using prepubertal rats, found ovarian degeneration in all animals receiving grafts, since the presence of necrotic and fibrotic areas was seen in the histology of the fragments, probably caused by tissue ischemia. In the present study, we found pyknosis, represented by condensed nuclei smaller than normal, with a lighter halo around the nucleus, a sign of cellular apoptosis (Santos *et al*., 2012). Oliveira (2011), also using rats as a model, found pyknosis in fresh and cryopreserved transplanted tissue, which corroborates the present study. In addition to the cryopreservation process itself, tissue manipulation can also cause damage.

## CONCLUSION

This study shows that autologous heterotopic ovarian transplantation is a feasible approach to testing the conditions of ovarian cryopreservation. We present a new and simple tool for ovarian vitrification, with promising results. Although our study, as well as all similar studies published so far, showed that there is significant tissue loss associated with the vitrification and transplantation procedures, cryopreservation of ovarian tissue remains an important alternative for some women. The development of more efficient cryopreservation techniques should be encouraged.
